# An Overview of Family and Community Nurse Specialists’ Employment Situation in Spain: A Qualitative Study

**DOI:** 10.3390/healthcare12222268

**Published:** 2024-11-14

**Authors:** Francisca Sánchez-Muñoz, Isabel María Fernández-Medina, María Isabel Ventura-Miranda, Ángela María Ortega-Galán, María del Mar Jiménez-Lasserrotte, María Dolores Ruíz-Fernández

**Affiliations:** 1 Department of Alicante, Health Care Centre San Vicente del Raspeig 1, 03690 Alicante, Spain; paquisanchezm9@hotmail.com; 2 Department of Nursing, Physiotherapy, and Medicine, University of Almería, 04120 Almería, Spain; mvm737@ual.es (M.I.V.-M.); mjl095@ual.es (M.d.M.J.-L.); mrf757@ual.es (M.D.R.-F.); 3 Department of Nursing, University of Huelva, 21071 Huelva, Spain; angela.ortega@denf.uhu.es; 4 Facultad de Ciencias de la Salud, Universidad Autónoma de Chile, Temuco 4780000, Chile

**Keywords:** primary care, family and community nurse specialist, experiences, qualitative study

## Abstract

**Background**: Family and Community Nurse specialists are advocates of a holistic model of care in multidisciplinary primary care teams. This study aims to describe the experiences and perceptions of nurses specialising in Family and Community Nursing regarding their working conditions in primary care in Spain. **Methods**: A qualitative descriptive study was conducted. Eighteen family and community specialist nurses from different autonomous communities in Spain participated. Individual interviews and a focus group were conducted. **Results:** The results identified two main themes: The current work situation of the Family and Community Nursing specialist and Support network and system of rejection with four sub-themes highlighting the lack of social and work recognition, the advantages of working with Family and Community Nursing specialists, systematic ambivalence towards Family and Community Nursing, and the need for institutional support. The inclusion of Family and Community Nursing specialists in primary care teams favours the nurse–patient bond, increases and/or maintains the quality of life of patients, and strengthens their empowerment; however, there is an absence of specific job vacancies. **Conclusions**: The institutional and social lack of awareness about the roles of Family and Community nurse practitioners and their impact on health care systems limits the quality of patient care in primary care.

## 1. Introduction

Family and Community Nurse Practitioners (FCNPs) are an important part of multidisciplinary primary care teams [1,2]. They are the driving force for change in a new model of integrated and holistic care centred on people, where health is promoted in different community settings [3]. These specialists must have in-depth knowledge of the complexity of individuals, families, and groups and the interactions that occur between them, as well as knowledge of the community in which they offer services [4]. The competences include care provision, public and community health, research, teaching, and care management [5].

At the international level, the development of the competency framework for FCNP specialists currently varies [1]. Thus, countries such as Australia, Canada, New Zealand, and the United Kingdom have developed competencies and/or national standards to clarify the role of FCNP specialists in primary care practice [6,7,8]. However, in other countries, there is no specific qualification or training for nurses working in primary care [1].

In Spain, the training programme for the speciality of Family and Community Nursing, which sets out the competences that these nurses must assume in multidisciplinary primary care teams, was approved and published on 17 June 2010 [4]. However, despite several years of training family and community specialists, the category is only recognised in 4 out of 17 autonomous communities in Spain [9,10]. The training period for FCNP specialists lasts two years [9]. Moreover, not all the country’s autonomous communities have created the category or defined the post of FCNP specialist [11]. Nevertheless, steps are being taken to ensure that the speciality of FCNP evolves and that FCNP specialists can develop their full potential in the primary care setting [3,12].

The integration of FCNP specialists into primary care teams has been associated with improved quality of care and their potential offers solutions to the changing health complexities and environments facing the health system, such as socio-economic determinants of health and population ageing [12]. Furthermore, more developed health systems have been linked to the trend of decreasing health care costs [13]. For this to happen, health systems must support the global advancement of nursing by supporting the speciality of FCNP through the incorporation of FCNP nurses in primary care settings [1,2,3]. Responding to these needs has the potential to facilitate their role in the delivery of community health care [14] as there is a great lack of knowledge on the part of the population and health organisations about the impact of FCNP specialists on the population’s health and well-being [15]. Few studies have analysed the experiences and perceptions of FCNP specialists once they are integrated into multidisciplinary primary care teams to differentiate their role [16,17]. Describing and understanding these experiences could be crucial to correctly describe their work situation, and the role they play in primary care teams, health institutions, and at the community level. This study aims to describe and analyse the work experiences of nurses specialising in Family and Community Nursing in primary care teams in Spain.

## 2. Materials and Methods

### 2.1. Design

A descriptive qualitative research design was followed [18]. This type of design allows us to reduce the researchers’ interpretation and focus on describing the participants’ experience in its natural state and from a closer perspective. Therefore, it will allow us to address the work experience of FCNP. The consolidated criteria of the COREQ guide were taken into account in reporting the findings of this study [19].

### 2.2. Participants

The study was conducted between January 2022 and December 2022. FCNP nurse specialists were invited through convenience sampling via email. Inclusion criteria were as follows: (1) to be a nurse specialist in family and community nursing; (2) to have completed specialist training within the last 5 years; and (3) to give consent to participate in the study. The exclusion criteria were as follows: (1) not replying to the study invitation email; and (2) being a specialist in family and community nursing by a route other than the resident nurse intern route. Finally, 32 participants replied to the email, of which 11 did not give their consent to participate in the study, and 3 did not attend the appointment on the day of the interview. A total of 18 nurses specialised in family and community nursing from various autonomous communities in Spain were included in the research. The most relevant characteristics of the participants can be found in Table 1.

### 2.3. Data Collection

First, a focus group was conducted which was supposed to be composed of 5 participants, but one of the participants did not show up. Therefore, the focus group was conducted with 4 participants. After the focus group, a total of 14 semi-structured individual interviews were conducted to explore some of the questions and issues raised during the focus group. Both focus groups and interviews were used to gain a more comprehensive understanding of the study phenomenon. Data saturation was reached with 12 participants and confirmed with the remaining participants [20]. The questions for the focus group and semi-structured interviews were developed after a literature review of the speciality training in different databases [1,5,12]. These questions were agreed upon and verified among the different researchers. The individual interviews lasted an average of 50 min. The duration of the focus group was 60 min. Both techniques were carried out on the Zoom platform due to the geographical dispersion. They were audio-recorded and then transcribed by the researchers with the consent of the participants. All participants offered to provide clarifications once the interviews had been transcribed. Appendix A shows the protocol and script of the interviews.

### 2.4. Data Analysis

The data analysis followed the guidelines of a reflective thematic analysis method Braun and Clarke [21]: (1) Familiarisation with the data: A thorough reading of the transcripts was carried out by the researchers to understand what the participants described; (2) Systematic data coding: The most significant quotes were selected and coded using the “live coding”, “open coding”, and “code code” functions in ATLAS. ti; (3) Generation of initial themes from the coded data: initial themes were generated by grouping codes with shared patterns of meaning, which related to a central idea; (4) Theme development and revision: all themes obtained, and the fragments on which they arose, were double-checked for consistency with the codes they included; (5) Precise, designate, and delimit themes: the researchers reviewed the final themes, refined them and fine-tuned the naming of each theme; (6) Report writing: the most demonstrative and eloquent quotations were selected. Finally, by filtering out the absolutely essential fragments and relating them to the literature review, the researchers clarified the report to ensure that the results were related to the objective of the study.

### 2.5. Ethical Issues

All ethical principles of the Declaration of Helsinki were respected [22]. All participants were informed of the aim of the study, the methodology employed, the voluntary nature of participation, and the possibility of leaving the study at any time without repercussions. Informed consent was obtained from all participants prior to the start of both the focus group and interviews. The confidentiality and anonymity of the participants was ensured through compliance with the Organic Law 3/2018, of 5 December, on Data Protection and Guarantee of Digital Rights. Authorisation was obtained from the Ethics and Research Committee of the Department of Nursing, Physiotherapy and Medicine of the University.

### 2.6. Rigour

To ensure methodological rigour, Lincoln and Guba’s quality criteria were respected [23]. Credibility was ensured by involving the researchers in the collection, analysis, and thorough description of the data collection process. In addition to this, the analytical process was carefully scrutinised by an independent reviewer. The transferability of the study has been maintained by thoroughly describing the method, participants, setting, and context of the study. Dependability was promoted by describing in as much detail as possible the methodology followed during the study. Reliability was maintained by the confirmation of a researcher who was not involved in the data collection and who corroborated the analysis conducted. Finally, confirmability was reinforced by the validation of the results by the participants.

## 3. Results

Two main themes and four sub-themes emerged from the data analysis, which allowed us to describe and understand the work experiences of Family and Community Nurse Practitioners in Spain (Table 2).

### 3.1. The Current Employment Situation of the Specialist in Family and Community Nursing

This topic shows the lack of job recognition of the speciality of family and community nursing, which hinders the provision of quality care in primary care. In addition, the changes that have occurred in recent years in the primary care system, together with the adverse effects caused by the COVID-19 pandemic, have made the applicability of the competencies of the specialist in FCN difficult. 

#### 3.1.1. The Lack of Social and Occupational Recognition of the Speciality of FCN

The participants in our study emphasised that despite the relevant role of the FCN speciality in the primary care system, there is a great lack of social and occupational recognition. The career path of the FCN specialists was marked by the lack of knowledge of the population and even of the health professionals themselves about their work and main competences, which caused great confusion and frustration in our participants.

*“The speciality of FCN is totally unknown to people. And it is not only unknown to people in the street, but also to many health professionals...”*.

(Participant 10)

This lack of recognition of the FCN specialist was related to the different barriers perceived at the employment level. The non-existence of employment contracts as specialist nurses, precarious salaries with hardly any salary recognition for the speciality, and the lack of job stability are barriers that hinder the development of the FCN speciality. In addition, the exclusive dedication to care activities or the hiring of specialist nurses as substitute staff hinders the provision and continuity of quality care.

*“It is easier to go to work as a generalist nurse in a hospital with more stability than to work in primary care”*.

(Participant 12)

*“We train as specialists even though we are paid a pittance to become better professionals and in the end it is not recognised”*.

(Participant 13)

However, most of the participants in our study indicated that they specialised in FCN because of the stability they could obtain in the world of work or because of the independence of working in your own practice. But the reality regarding the world of work is quite the opposite of what they thought before specialising.

*“Well, I chose it [referring to the speciality] because of the job stability it would give me and because of the independence that a nurse has in his or her own practice”*.

(Participant 11)

The current COVID-19 pandemic has had a strong influence on the FCN speciality. Some participants felt that the pandemic has facilitated the recruitment of nurses in health care facilities, but this increase has not been due to the fact of having a specialist degree in FCN but to the urgent need for nurses. Even so, our participants reported that the pandemic has made the work in primary care more difficult. It meant a drastic change in the organisation and in the way of caring for patients and families, which has meant a setback in primary care. The primary functions of the FCN nurse, such as health education, community activities, and chronic patient follow-up, have been eliminated.

*“Even though I have the speciality, if there was no pandemic I don’t think I would have been able to work in primary care”*.

(Participant 7)

*“Primary care has been destroyed with the pandemic, it is no longer primary care, many functions of nurses have been lost and it will be difficult to recover them”*.

(Participant 10)

#### 3.1.2. The Advantages of Working with FCNP Specialists

The participants in our study explained that due to their training in primary care, the management of their competences as FCNP specialists favours the increase or maintenance of the health of the population, contributing to the creation of a healthier and more empowered population. All participants agreed on the power of FCNP specialists to communicate and transmit knowledge to their patients to help them improve their health situation.

*“I think prevention and health promotion is a wonderful thing! But even though I love my work and continue to strive to be trained, it is not recognised”*.

(Participant 6)

According to the opinion of our participants, specialisation increased their self-confidence and self-assurance, helped them to have a more holistic view of the patient, and favoured the development of their empathy skills. The nurses perceived that the creation of a network of nurse-patient-family trust favours the empowerment of the users, increases the effectiveness of their interventions, and decreases over-frequency in the consultations. This opinion was also shared by the patients and colleagues themselves, who consider the FCNP specialists to be the team’s reference persons.

*“Patients are delighted to have a specialist nurse because they realise that the treatment and quality of care is much better”*.

(Focus group)

*“Resolute girl, all-rounder girl..., this was also said to other specialist colleagues”*.

(Focus group)

All the participants reported that they were saddened by the fact that the public administration did not encourage the development of the competences of FCNP specialists despite the numerous advantages offered by the nursing specialisation. They feel that they are a professional group that is underutilised and heading towards involution, so they are currently fighting against the tide for the recognition of the FCNP speciality.

*“You always have to be fighting for your place as a specialist nurse”*.

(Participant 6)

*“We have gone backwards despite having specialist nurses and the population does not feel that we are on their side taking care of them”*.

(Focus group)

### 3.2. Support Network and Rejection System for FCNP

This theme includes both the support received and the barriers encountered by FCNP specialists during their working life. Our participants insist on the invisibility of specialist nurses and that, because they are currently a minority, they do not receive sufficient support to be able to evolve. 

#### 3.2.1. Systematic Ambivalence Towards FCNP

Nurse specialisation is fundamental to the provision of quality care and to the transformation of the health care system. The participants in our study described the support they received from family and friends as well as from colleagues and nursing management in the primary care centres where they worked. The FCNP specialists are seen as leading professionals in the implementation of health education projects, community interventions, and mentoring of undergraduate nursing students and nurses in training in FCNP. However, they point out that this support perceived by the nursing management is hindered by the barriers that the public administration puts in the way of the development of FCNP.

*“The work contract is not for a specialist...this figure does not legally exist yet because it is not recognised, but they [referring to nursing management] did want a specialist to cover it”*.

(Participant 15)

However, some of the participants described feeling rejected by some colleagues. Derogatory comments about the FCNP speciality and comparison with non-specialists made our participants feel uncomfortable, undervalued, and in some cases excluded. They even felt that their colleagues were suspicious, envious, or afraid of them for being an FCNP specialist.

*“From colleagues I have noticed anything from indifference to reticence, and sometimes even contemptuous treatment”*.

(Focus group)

*“The main enemy of the nurse is the nurse himself”*.

(Participant 7)

#### 3.2.2. Need for Institutional Support for FCNP

Our participants expressed the lack of institutional support for FCNP specialists throughout the study, especially highlighting the precarious salaries, job instability, the virtual non-existence of job exchanges, and specific competitive examinations for FCNP specialists. They detail obstacles in recruitment, such as the lower score for time worked during the training period or as an FCNP specialist in the general nursing pool.

*“The time spent training or working as an FCNP specialist scores you half as much in the general nursing pool. It seems that we are being punished instead of being rewarded”*.

(Participant 7)

This job insecurity slows down the evolution of the speciality and hinders nursing leadership. The participants in our study explained that the public administration should give priority to FCNP specialists not only for recruitment in primary care but also for management and leadership positions. In addition, the creation of specific job vacancies and competitive examinations for FCNP specialists would favour job stability and the full development of all their competences.

*“I think we should be allowed more access to management positions, and by that I mean school management”*.

(Participant 15)

*“I have the feeling that I am not doing as much as I could... and on the other hand I feel that I cannot grow in the profession without the support of the administration”*.

(Participant 8)

Support from trade unions and nursing associations was also not perceived by the specialist nurses in our study. They all felt extremely disappointed with these bodies, commenting that the help received has been practically nil and that they have even felt rejected and excluded when they demanded recognition of the speciality before these bodies.

*“We are paying dues to the nursing union or college that do not contribute anything... I left [referring to the nursing union] because they did not support the specialities”*.

(Participant 12)

This weak recognition of the FCNP speciality caused our participants to feel that they were unnoticed health professionals. They urgently call for support for the FCNP speciality, so that the nursing profession can progress, and the population can benefit from all its advantages.

*“As specialists we don’t exist, nobody fights for us to be there [for the community], except ourselves”*.

(Participant 10)

## 4. Discussion

This study aimed to explore the perceptions and experiences of nurses specialising in primary care in Spain, with a particular focus on their working conditions. The family and community nurse specialists in our study reported that there is a lack of job recognition by the health system. This lack of job recognition was related to the lack of knowledge of the role of the specialist nurse both at a general level and at the level of the health system with the health professionals themselves [6,8,17]. In line with our results, different authors have highlighted at the international level the lack of consensus on the definition of the designation of FCNP and the need for further progress on this issue in order to make its role visible to society and health administrations [1,24]. The participants in our study mentioned that they consider that the health system does not seek the evolution of nursing, as they have encountered numerous barriers in order to be able to enter the labour market as FCNP specialists. This fact is reported in the study by Miguélez Chamorro and Ferrer Arnedo [25] where they described the need to analyse and correct the factors that limit this professional growth, including the lack of definition of competences and the heterogeneity of the profession. In addition, they described that on many occasions they felt used due to their commitment to the speciality and the population and that the administration took advantage of this commitment and their vocation at some point. These perceptions cannot be compared with other articles as there are no studies on these type of feelings in nursing. Therefore, the results of this qualitative study give an insight into the feelings of the nurses during their specialisation and how their motivation was frustrated by a health system that was not in line with the competences they could develop.

However, we have found that despite their heavy workload, they feel motivated and committed to the population [2,26]. They also described that, in certain situations, they felt underutilised and that they could not carry out all their competencies because they did not have the resources, including not having an FCNP contract [10]. In fact, all of them, as well as several authors, described the benefits of the competencies provided by FCNPs, highlighting their great potential to favour the health of the community, the relationship of trust with both the patient and their families and the influence they exert on the health empowerment of patients [1,8,27,28]. They also consider it imperative that the practice of their training and competencies be facilitated as much as possible to optimise the efficiency and quality of services to patients [29]. Likewise, our participants highlighted the importance of their role in education, health promotion, and community activities, considering their work in this area indispensable [28,30].

Similarly, our participants also proudly described the power they possess to communicate and transmit knowledge both to their patients and to other members of the primary care team, relating this to their leadership skills [31]. However, the effects of the current COVID-19 pandemic could not go unmentioned, they mentioned that, despite increasing the recruitment of nurses, they had negatively influenced the application of the competences of the FCNP specialist, and thus, the continuity of care for users. Among the skills that were lost were the decline in health education, community activities, and chronic patient follow-up. These findings were described in the article by Pingel et al. [32], where they mentioned that the COVID-19 pandemic has meant a decrease in the performance of basic primary care competences, such as prevention and health promotion activities. In addition, they mentioned that they had followed up on users who contracted the disease but were unable to continue with the continuity of care for the rest of the users, a situation that our participants currently feel that the system is not helping enough to recover. This is echoed in the study by Ares-Blanco et al. [33], where we can see the need for a strategic plan for primary care in Europe to help achieve this challenge. In this plan, they consider the need to strengthen continuity of care and coordination between levels.

Finally, all the participants described feeling supported by their family and friends, by their colleagues, by most of the nursing directorates, and by the EFyC scientific associations. On the other hand, they did not perceive the same support from the Nursing Associations and especially from trade union groups, where most of the time, they felt displaced. These facts cannot be compared with other research due to the lack of such studies. Only the study by Lukewich et al. [2] emphasised the perceptions of Norwegian nurses who described feeling invisible to their work support system. Perhaps this is because they are considered a minority group compared to generalist nurses working in health systems, and therefore, they do not consider themselves represented by these organisations.

### 4.1. Limitations

Firstly, the participants were FCNP specialist trained only in the communities of Castilla-La Mancha, Comunidad Valenciana, Girona, Murcia, and Madrid due to the provision of FCNP in these communities. Moreover, these regions have specific characteristics such as differences in primary care management that could influence the results. Even so, it has been possible to know the employment situation of FCNP specialists in Spain. Secondly, all the participants were women, so the perspective may influence the results. However, this is a reality rather than a limitation, since the nursing profession is primarily a female profession. Finally, the different data collection techniques were carried out in online format and non-verbal language was not clearly visible, but the scientific rigour used gave credibility to the research.

### 4.2. Future Lines of Research

Future research could describe the perceptions of users, co-workers, and coordinators and/or directors of nursing about nurses specialising in FCNP, in order to raise awareness of the role of the nurse specialising in FCN and to help health systems to make the most of this human resource and to promote its recognition in the workplace. In addition, a longitudinal study to observe how barriers and perceptions of FCNPs evolve, especially in post-pandemic contexts, would be useful to assess their impact on the care of the population.

## 5. Conclusions

The results of this study indicate that the inclusion of FCNP specialists in primary care teams favours the nurse–patient bond, increases and/or maintains the quality of life of patients, and strengthens their empowerment. The FCNP specialists are professionals who serve as a point of reference for knowledge in primary care for their colleagues. Despite this, there is a great lack of awareness of their role both in the workplace and in the population. The absence of specific job vacancies for FCNP specialists or the lack of job stability prevents them from carrying out their competencies with the quality for which they are qualified. There is a clear lack of employment support for the FCNP speciality which may affect the quality of care provided to patients.

It is necessary to continue working on unifying the role of the FCNP specialist in order to facilitate their incorporation into primary care. The incorporation of specialist nurses in other countries such as Canada and Australia has led to clear advances in health outcomes at the individual, family, and community levels. For this reason, it is considered important to create and activate specific job exchanges and competitive examinations for FCNP, to give importance to nursing leadership by encouraging FCNP specialists to occupy nursing management and leadership positions, and to provide financial incentives for the efforts made by FCNP specialists in their training. On the other hand, it is necessary to make society aware of the role of the FCNP and the population in health care and health promotion. To this end, awareness campaigns should be carried out by the health administrations and the scientific societies that include these nurses.

## Figures and Tables

**Table 1:** Socio-demographic characteristics of the participants.

Participants	Source of Data	Age	Sex	Autonomous Community Training	Year of Completion of Speciality	Time Working in Primary Care
1	Focus group	32	Woman	Madrid	2020	1 year and 9 months
2	Focus group	35	Woman	Madrid	2020	1 year and 9 months
3	Focus group	51	Woman	Madrid	2020	1 year and 9 months
4	Focus group	27	Woman	Madrid	2020	1 year and 9 months
5	Interview	27	Woman	Madrid	2021	9 months
6	Interview	25	Woman	Madrid	2021	1 year
7	Interview	26	Woman	Castilla-La Mancha	2020	1 year and 8 months
8	Interview	28	Woman	Madrid	2019	4 years
9	Interview	26	Woman	Castilla-La Mancha	2021	2 years
10	Interview	31	Woman	Madrid	2018	3 years
11	Interview	27	Woman	Madrid	2020	7 months
12	Interview	28	Woman	Madrid	2018	1 year
13	Interview	31	Woman	Madrid	2019	2 years
14	Interview	27	Woman	Catalonia	2021	2 years
15	Interview	28	Woman	Madrid	2018	3 years and 6 months
16	Interview	29	Woman	Madrid	2019	3 years and 6 months
17	Interview	28	Woman	Murcia	2020	7 months
18	Interview	33	Woman	Valencian Community	2018	2 years and 6 months

**Table 2:** Themes, sub-themes, and units of meaning.

3.1. The current employment situation of the family and community nurse specialist.	3.1.1. The lack of social and professional recognition of the family and community speciality.	Lack of knowledge, lack of recognition, specific job opportunities for FCNS, precarious pay, employment contracts, COVID-19.
	3.1.2. The advantages of working with family and community nurse specialists.	Health education, confidence and security, quality care, increased knowledge, essential work.
3.2. The family and community nurse specialists’ perceptions of support and rejection.	3.2.1. Systematic ambivalence towards family and community nurse specialists.	Family and social support, professional support, job rejection.
	3.2.2. The need for institutional support for family and community nurse specialists.	Lack of professional and employment support, minority, invisibility.

## Data Availability

Data are contained within the article.

## Appendix A

**Table A1:** Examples of questions used in the interviews.

Phase	Development	Content/Example Question
Beginning of the interview	Motives	As family and community nurse specialists, we would like to hear about your work experiences in recent years.
	Ethical issues	Participation is entirely voluntary and you are free to withdraw from the study at any time. The sessions will be recorded and subsequently transcribed. Your identity will be protected and your personal details will not be disclosed.
	Introductory question	What is your experience as a nurse in family and community nursing? And specifically, in the last few years?
Conducting the interview	Conversation guide	Do you think that the family and community nursing speciality has both professional and personal advantages? Why? What kind of difficulties have you encountered seeking work as a family and community nurse specialist? What do you think about this? Do you feel satisfied in your workplace? Why is this the case? How do you think the work situation of family and community nurse specialists could be improved? Do you feel supported by the ‘system’, trade unions, nursing associations, etc. as a family and community nurse specialist? On what basis do you answer this question?
	Final question	Is there anything else you would like to add to this interview that you think is important and that we have not mentioned?
Closing the interview	Acknowledgements	Thank you very much for your participation and for dedicating your time to this research. Your input is very important to the study and will be very helpful.
	Contributions	We would like to remind you that you are welcome to contact us if you have anything else you wish to add at a later stage. After the transcription process of the interviews has been completed, we will show them to you, and once the study has been completed, we will show you the results.
